# Exploring the evidence base for how people with dementia and their informal carers manage their medication in the community: a mixed studies review

**DOI:** 10.1186/s12877-017-0638-6

**Published:** 2017-10-18

**Authors:** Lydia Aston, Andrea Hilton, Tiago Moutela, Rachel Shaw, Ian Maidment

**Affiliations:** 1 School of Life & Health Sciences, Aston University, Birmingham, UK; 2 Faculty of Health Sciences, University of Hull, Hull, UK; 3 School of Life and Health Sciences, Aston Research Centre for Healthy Ageing (ARCHA) and Pharmacy Department, Aston University, Birmingham, UK

**Keywords:** Dementia, Medicines management, Community, Informal carers, Healthcare professionals

## Abstract

**Background:**

Little is known about the general medicines management issues for people with dementia living in the community. This review has three aims: firstly to explore and evaluate the international literature on how people with dementia manage medication; assess understanding of medicines management from an informal carers perspective; and lastly to understand the role that healthcare professionals play in assisting this population with medicines management.

**Methods:**

A mixed studies review was conducted. Web of Knowledge, PubMed and Cochrane Library were searched post-1999 for studies that explored medicines management in people with dementia dwelling in the community, and the role healthcare professionals play in supporting medicines management in people with dementia. Following screening, nine articles were included. Data from included studies were synthesised using a convergent synthesis approach and analysed thematically to combine findings from studies using a range of methods (qualitative, quantitative and mixed methods).

**Results:**

Four themes were generated from the synthesis: The nature of the disease and the effects this had on medicines management; the additional responsibilities informal carers have; informal caregivers’ knowledge of the importance of managing medication and healthcare professionals’ understanding of medicines management in people with dementia. Consequently, these were found to affect management of medication, in particular adherence to medication.

**Conclusions:**

This review has identified that managing medication for people with dementia dwelling in the community is a complex task with a frequently associated burden on their informal caregivers. Healthcare professionals can be unaware of this burden. The findings warrant the need for healthcare professionals to undergo further training in supporting medicines management for people with dementia in their own homes.

## Background

Dementia has an estimated global prevalence of over 35 million people and affects over 0.5% of the global population with annual societal costs of $604 billion [1]. As the disease progresses, people with dementia are at risk of developing medication related problems and becoming non-adherent to prescribed regimens [2–4]. This can be caused by a number of factors, such as inadequate knowledge regarding medication, which may result in incorrect doses or administering the treatment at an inappropriate time of the day [5]. Additionally, issues with medication can occur if an individual exceeds the required amount or if mistakes are made in the prescribing, dispensing or administration process [5, 6]. The risks associated with medication can proliferate if the person with dementia is on a complex medication regimen due to co-morbid conditions [5, 7]. Medicines management and issues with medication is an international problem that is not just restricted to the UK [7–9].

The two most commonly used terms to describe the process of safe and effective medicines use are ‘medicines management’ and ‘medicines optimisation’. Medicines management is defined as: “the entire way that medicines are selected, procured, delivered, prescribed, administered and reviewed to optimise the contribution that medicines make to producing informed and desired outcomes of patient care” [10]. The relative new term of medicine optimisation is defined as “a person-centred approach for the most safe and effective use of medication to ensure the best possible outcome for the patient” [11]. The term medicines management has been in use longer and is used throughout this article to capture a broader chronological range of articles.

Effective medicines management requires the involvement the person with dementia, the informal carer and multiple healthcare professionals [12]. In order to gain a full and meaningful appreciation of medicines management, it is arguably important to address all three points of view. To date, much research has focused on the stress and coping techniques found in caring for someone with dementia without reference to the particular impact of medication and its management [13]. Other research has presented a significant but partial view: either focusing on the perspective of the person with dementia and their carer as part of a more general review [8] or focused on the challenges of medicines management across both the community and care home settings without the perspective of healthcare professionals as part of this [14].

It is important to understand medicines management in the community as a setting reliant on the interdependent relationships between all three strands of the equation, as the person with dementia, informal carers (family or friends with an unpaid caring role), and healthcare professionals attempt to engage with this issue effectively. To our knowledge, no studies have sought to assess and appraise research that investigates the tripartite relationship of the person with dementia, their informal carer and healthcare professionals. Establishing a good understanding of this whilst people are living in the community may help to identify methods of early detection and intervention to continue care at home, with regards to managing medication and may prevent or delay hospitalisation and care home admittance.

This review aimed firstly to explore and evaluate published international literature on understanding how people with dementia manage medication whilst living in the community. Secondly, it sought to gain understanding of what is known about the effect of managing care-recipients’ medication on informal carers. Thirdly, the way in which healthcare professionals support people with dementia and their informal caregivers with medicines management was assessed.

## Methods

A mixed studies review methodology was adopted, which enabled the consideration of contextual factors in the investigation of medicines management in people with dementia [15].

### Data sources

PubMed, Web of Science and Cochrane Library were initially searched in November 2014 with restrictions to English and post 1999. Searches were updated several times and a final search was completed in January 2017 (with two further papers being included).

### Search strategy

Consultations with an information specialist and IM and AH were used to generate preliminary search terms to see if this resulted in relevant articles being found. This iterative approach developed and refined the search terms. The search strategy is included in Table 1. LA conducted the search and evaluated potentially relevant articles to include in this review. The list of identified studies was independently assessed by IM. Any disagreements about inclusion were discussed and resolved by consensus by LA and IM.

**Table 1:** Search strategy

Topic	Search terms
Medication	“dementia” OR “alzheimers disease” AND “caregiver” OR “carer” OR “family carer” OR “informal carer”

### Inclusion criteria

Studies included:

- Were set in the community, in the homes of people with a diagnosis of dementia.
- Primary research only - using any design method including randomised controlled trials, intervention studies and studies using quantitative, qualitative and mixed methods data.
- Full text papers.
- Covered the perspective of either the person with dementia, informal caregiver to someone with dementia and/or healthcare professionals regarding medicines management in people with dementia in the community.

### Exclusion criteria

- Focussed on elderly people or other conditions as well as dementia i.e. schizophrenia/Parkinson’s Disease (PD), thereby lacking specificity.
- Care home settings.
- Involved end-of-life care.
- Focussed on the treatment of Behavioural and Psychological Symptoms of Dementia (BPSD), complimentary medicine or pharmacology of specific drugs rather than general medicine management.
- Involved specific medications without a focus on medicine management.
- Non-English language studies (translation facilities were not available).
- Pilot studies and secondary analysis papers such as literature reviews were excluded.

### Critical appraisal

LA, AH and TM independently assessed the study quality of the included papers using the Mixed Methods Appraisal Tool (MMAT) [15]. This tool is purposely designed to appraise the methodological quality of studies that use a range of methods [15]. The MMAT is a validated checklist, providing a set of criteria for screening questions, which are applied across the included studies, providing a score for each study. Adopting the MMAT meant that it was possible to appraise studies with different methodological designs using the same tool [16]. LA, AH and TM discussed the quality ratings of the included studies based on the MMAT and agreed, by iteration, on the final quality score for each.

### Data extraction and synthesis

A convergent synthesis approach was adopted using thematic analysis [16]. All data were extracted into the same file: qualitative data were copied verbatim; quantitative data presented numerically in tables or figures were summarised in words sometimes using interpretations from the discussion sections of papers. Extracted qualitative and quantitative data comprise both verbatim extracts from participant accounts (in the case of qualitative work) and verbatim extracts from authors’ interpretations (in the case of qualitative research from the results section and in quantitative papers from the discussion section). It is commonplace for syntheses of this nature to include as data, therefore, extracts from authors’ interpretative analyses because this is a secondary analysis of included studies [17].

The file containing the extracted data was presented as a matrix organised by studies and themes identified in the results of included studies. This matrix was then searched to identify commonalities across papers. This process was similar to the constant comparison technique often used in grounded theory and involved highlighting data that were comparable across studies and coding them into categories which represent those common themes [18–20].

Following that, an interpretative thematic analysis took place, informed by the thematic analysis of primary data [21]. This meant looking at the common themes identified and exploring their meaning in relation to the review question: we asked what each category told us about the phenomenon of medicines management in the community among people with dementia. This process is referred to as a convergent synthesis, which means that all data are taken together, interpreted, and represented as a set of themes developed through an interpretative secondary analysis [16]. LA led the convergent synthesis and discussed the emerging themes with IM. LA and IM discussed and further developed the themes and agreed the final set.

## Results

### Study selection

**Fig. 1:**
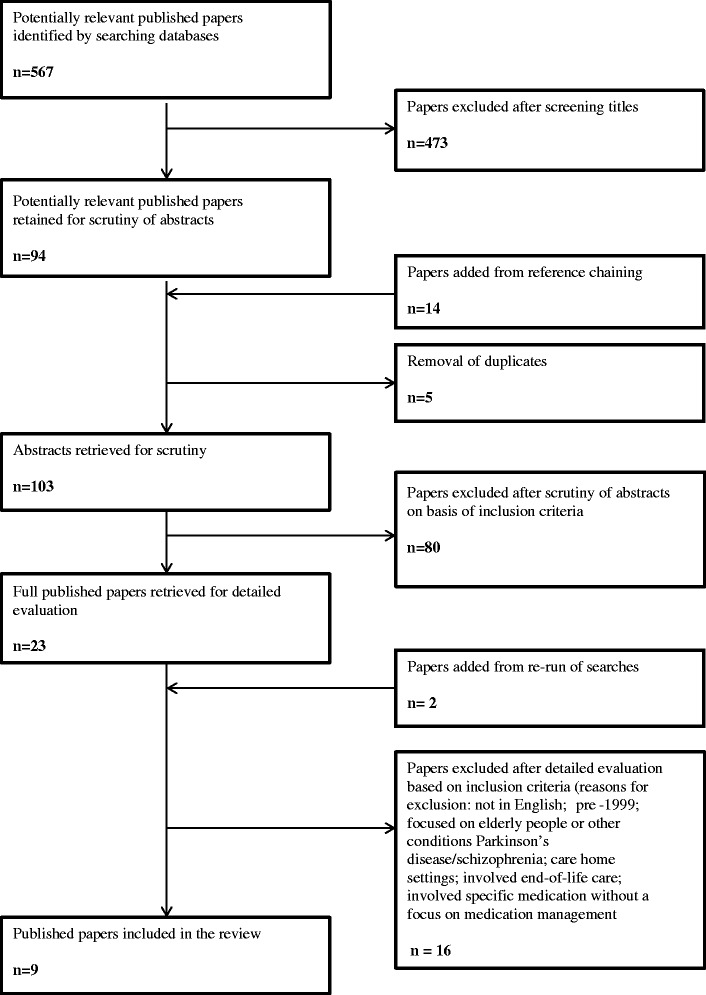
PRISMA flowchart

The search yielded 567 references, which were screened in accordance to the inclusion criteria (see Fig. 1). This was followed by “reference chaining” of those studies included. Referencing chaining in this context involved looking at the references that papers included in the study had used. These were then screened by title to determine if there were any further papers deemed suitable for further reading. Papers were screened by title and abstract; any potential papers for inclusion were read fully before including or excluding. Searches were re-run several times throughout the research period to ensure no new literature was published, the final search was January 2017. Three papers focused on medicines management in people with dementia and informal carers [12, 22, 23], two papers focused purely on informal carers [24, 25], one solely on people with dementia [26], two combining people with dementia, informal carers and healthcare professionals [27, 28] and one paper explored attitudes of community pharmacists towards people with dementia [29]. Nine studies were included in the review (see Table 2).

**Table 2:** Characteristics of included studies

First author & date	Journal	Aim	Sampling Method	*n* =	Location	Data collection	Analysis	Quality rating
Barry (2013)	International Journal of Geriatric Psychiatry, 28, 1077-1085.	To explore community pharmacists’ experiences with and attitudes towards people with dementia (focussed on pain).	Purposive	Community pharmacists: 182	Northern Ireland	Questionnaire	Descriptive analyses and Chi-square and Fisher’s exact test	50%
Fiss (2013)	International Journal of Geriatric Psychiatry, 28, 173-181.	To analyse the occurrence of Potentially Inappropriate Medication (PIM) taken by elderly individuals in the AGnES studies in primary care.	Convenience	Patients: 342	Germany	German PRISCUS list	Phi coefficient and multiple binary logistic regression analysis	75%
Gillespie (2015)	Dementia, 14, 47-62.	The study aimed to explore the views of ethnic minority informal caregivers of people living with dementia and their medicines management experience including the adequacy of their access to medication management information and support.	Purposive	Caregivers: 29	Australia	Focus groups and individual interviews	Thematic analysis	50%
Kaasalainen (2011)	Journal of Nursing and Healthcare of Chronic Illness, 3, 407-418.	The purpose of this grounded theory study was to explore the personal experiences related to medicines management of community-dwelling older adults diagnosed with dementia, their informal caregivers and healthcare professionals who assist them.	Theoretical sampling	Community health nurses: 10 Pharmacists: 10 Family Physicians: 6 Informal caregivers: 20 People with dementia: 11	Canada	Interviews	Grounded theory	50%
Maidment (2017)	Health Expectations, 20, 929-942.	To describe and understand the key challenges, in relation to medication issues, experienced by people with dementia and their informal carers dwelling in the community and the potential role of community pharmacists	Purposive and snowball effect	Informal carers: 11 GPs: 4 Nurses: 5 Social care professionals: 3 Community pharmacists: 4 People with dementia: 4	UK	Interviews	Framework analysis	75%
McKenzie (2013)	American Journal of Alzheimer’s Disease & other Dementias, 28, 348-354.	The primary aim of this article is to report the implementation and adoption of the Safe Home Program and the caregiver assessments of these technologies and devices to determine which one may be used to support caregivers.	Purposive	People with dementia: 60	USA	Questionnaires and interviews	Not specified	75%
Poland (2014)	BMC Research Notes, 7, 463.	The paper aims primarily to describe the Public Patient Involvement process which was intended to inform the development of a future research proposal	Purposive	Carers: 9	UK	Focus group	Thematic analysis	100%
Smith (2014)	International Journal of Pharmacy Practice, 23, 44-51.	The aims of this study were to examine the scope and range of medicines related assistance provided by informal carers of people with dementia, the problems that arise and to identify how service provision could become more responsive to these needs.	Convenience	Carers: 14 Care-recipients: 5	UK	Interviews	A framework analysis	75%
While (2012)	Dementia, 12, 734-750.	This paper examines the perspectives of the person with dementia and their carers to explore if there are any significant differences in their medicines management experiences when compared to older adults without dementia and their carers.	Purposive and snowballing approach	People with dementia: 8 Informal carers: 9	Australia	Interviews	Thematic data analysis and critical analysis	75%

### Study quality

The critical appraisal (MMAT) of the included studies showed a variation in their quality (see Table 2). Consideration for contextual influences and researchers’ influence on the research conduct was lacking [12, 23, 24, 27]. Transparency of recruitment procedures was lacking in one paper [26]. The quality of included studies varied (a summary quality is in Table 2). Papers judged to be poorer quality were not excluded as it was still important to include their findings. However, studies rated to be higher in quality were given greater ‘weight’, in that these papers were discussed more in the synthesis [17].

### Study characteristics

Six studies used qualitative methods [12, 23–25, 27, 28], two used quantitative methods [26, 29], and one used a mixed methods design [22]. Four studies were conducted in the UK and Northern Ireland [23, 25, 28, 29], two in Australia [12, 24], one in the USA [22], one in Canada [27] and one in Germany [26] (see Table 2).

### Synthesis

The results of included studies generated four themes:

1. Effects of dementia on medicines management and the use of adherence aids
2. Impact on informal carers
3. Knowledge of medication as an aid to adherence
4. Healthcare professionals’ understanding of medicines management in people with dementia

#### Effects of dementia on medicines management and the use of adherence aids

With any increasing severity of dementia adherence became more challenging [12, 24, 29]. Informal carers commented that as their care-recipients no longer understood the importance of taking medication this could result in non-adherence. “*My husband he remembers, he takes his own medication what [sic] he like but he only takes the blood pressure tablets because the other tablets he thinks do not do anything to him so it’s no worth to take it*” [24]. This calculation of whether medication makes a difference to the person with dementia is portrayed here, which affected adhering to prescribed medication. This increased risk in non-adherence to medication occurred even when people with dementia have informal carers present [29]. One study found people with dementia received more support with drug administration compared to those without dementia *“dementia: 72.0%; no dementia: 36.8%”* [26].

The determination of people with dementia to continue to manage their own medication was found in five studies [12, 23, 24, 27, 28]. People with dementia wanted to *“develop and maintain an ability to remember their regime”* [23] and some informal carers appeared to support their autonomy [24]. Nevertheless, due to the progressive nature of dementia, the person’s ability to remember to take and manage their medication was an issue that was commented upon in over half of the included studies [12, 23, 26–28]. “*At first she was taking them every time and then it sort of degraded… She was getting worse as time was going on”* [12].

Some people with dementia recognised a change in their own cognitive ability to manage their medication which seemed to cause them distress: “*He was very defiant about the fact that he had taken his medication and then he was very embarrassed when he found he hadn’t”* [12]. However, others seemed more accepting of their informal carers being involved: *“Well my family need to know all about it… Because there is no point telling me, so they have to know everything”* [23] thus presenting a difference in coping strategies of living with dementia.

Adherence packs were commented upon by informal carers. Some informal carers organised for the adherence packs to be made up by their pharmacist and these appeared to initially help in the early stages of dementia or had worked for a time but as the disease progressed and further cognitive decline occurred, these aids became less helpful [24]. In order to help reduce non-adherence, one study implemented a ‘safe home programme’ where informal carers of people with dementia were provided with a medication organiser device if the care-recipients were on a complex medication regime (>7 medications) [22]. This device could be set to give alarms to provide an alert to the informal carers when medication was due. Informal carers found this device useful as it gave them *“…the ability to set up a month’s supply of medicine”* [22]. Based on the studies included in this review, this may act as a useful tool for people with dementia in the early stages of dementia. However, it may be too complex, for usage, when the severity of dementia increases.

Some studies found people with dementia were resistant to change and utilise medication aids as they did not want to form a new routine: *“My sister and I, we discussed eventually we will arrange blister packs for her but she really doesn’t want to do that... she likes things the way she’s always done them”* [24]. This resistance to change could result in conflict between the care recipient and carer as discussed in theme 2. However, it might also signify that there are other issues that need to be considered such as stigma towards dementia, loss of sense of self and pride when ‘failures’ of this kind are noticed. Interventions should take into account the many transitions that people with dementia and their informal carers have to go through. The desire of people with dementia to hold on to something that is known, although it may be more difficult, was found to sometimes be preferable, reflecting a need for control in organising the medication themselves [23].

#### Impact on Carers

As reported in one of the included articles, changes to the prescription and the use of self-filled adherence aids increased the burden and carer stress “*It meant I had to make this box every week*” and “*But it would be so much easier if things would go automatically*” [23]. This was shared in another study where carers commented on the difficulty of keeping on top of multiple medications, which in turn meant that they would have to visit the pharmacy “*twice a week*” [28]. This illustrates the critical role that informal carers have in supporting medicines management. People with dementia also reported the need to avoid changing the regimen in order to keep things simple, “*Something new added can really throw my whole routine off…So if things are simple … it’s easier*” [27].

A study conducted in 2012 found that carers particularly struggled with the role when they first took it over “*Well, this caused me a lot of burden and stress in the beginning*.” [12]. They reported that a lack of cooperation from the person receiving support and any inconsistency in obtaining medication increased the burden [12]. Yet another study found that carers would prioritise their care recipient’s health above their own *“Sometimes I feel fed up but what can I do? That is my duty…I forget my medicine but I never forget his.”* [28]. Feeling isolated without adequate support from healthcare professionals also increased the burden [24, 25, 28]. Receiving better support from healthcare professionals may help to alleviate a part of the burden that informal carers feel, providing them with better provision to help their care-recipient.

Anxiety about their competence in the role increased the burden “*I really do wish you wouldn’t ask me how I’m coping because the word coping implies that if I’m not, it’s my fault*” [25]. This may be related to issues such as the stigma of living with and caring for someone with dementia, due to the lack of public awareness and understanding of the disease. It is also wrapped up within a deep sense of responsibility that the carer feels in having to care for someone with dementia in the ‘accepted’ way. Carers found complex regimens confusing “*And he was put on further more tablets. I don’t know what they are for or what they are*” [24]. The transition between the person with dementia self-medicating and medication being managed by the informal carer could be particularly stressful. The informal carer needs to make a judgement that the person with dementia can no longer safely manage their medication, inform the person and take control. This need to balance safety with a person-centred approach can result in conflict. “*He said ‘what do you think!? Do you think I can’t manage medications!?*’” [24].

#### Knowledge of medication as an aid to adherence

A number of papers found that informal carers sought to understand the key aspects of their care-recipient’s medication [23–25]. This again is connected to the increased dependence on informal carers. Many informal carers across the studies discussed how they actively sought information about medicines and their side effects: [23–25] “*They (informal carers) described reading package information, researching on the internet, magazines, telephone calls to a doctor and two carers had access to a British National Formulary (BNF)*” [23].

Another study found that some informal caregivers were concerned about the effects that the medication was having on their loved ones, and questioned the benefits of taking it [28]. Informal carers were shown to question the decision of the healthcare professionals in the medication that they had prescribed for their care-recipient; this was linked to an increased risk of non-adherence. One reason for this may have been due to the lack of difference they saw in their care-recipients, which initiated questions as to the usefulness of the medication prescribed: *“I need to see Dr X, I really do…the tablets that she's on, they are not doing anything. I often wonder…to experiment and not give her any tablets at all for a week and see what the outcome would be”* [28]. This assumed responsibility for determining whether the medication was working and the amount that should be given was found in the next quote: *“[Doctor said] Every three days, but I don’t give it to her every three days because it’s a morphine patch, it’s for pain. She isn’t saying that she’s...any pain. Following the prescription she’d be taking 8 paracetamol a day which I think is far too much to be honest with you”* [28]*.* This self-appointed understanding of what was best for the person with dementia was acknowledged and may demonstrate the lack of opportunity informal carers felt they had in communicating problems with healthcare professionals, thus presenting a barrier to effective medicines management (see theme 4).

One study deemed to be of high quality based on the MMAT, found that informal carers wanted healthcare professionals to provide them with a *“checklist”* that presented basic information in plain language regarding the medication that their care-recipients were taking; this should include *“…their (medication) effects, side effects and usage instructions”* [25]. This suggests that greater understanding and knowledge about medication is valuable to informal carers and in turn may help to encourage optimal medicines management.

#### Healthcare professionals’ understanding of medicines management in people with dementia

Carers reported that Health or Social Care Professionals may be unaware of this burden and the challenge associated with medicines management [12, 28], including practical daily issues “*Don’t forget that the clinician and pharmacist can have little or no understanding of the practicalities”* [25] yet a key part of the healthcare professional’s role is dementia care. One study sent questionnaires to pharmacists about their involvement with people with dementia, specifically regarding their knowledge about the management of pain in this population; they found that: “…*nearly all respondents (91.2%) had provided pharmaceutical care to people with dementia living in their own homes”* [29] and most would often support the person with dementia’s informal carers. However, findings suggest that pharmacists showed uncertainty in treating people with dementia, in regards to assessing and treating pain. This was highlighted in the: “*large proportion of respondents who chose to ‘neither agree nor disagree’ with certain statements”* [29]. The majority of pharmacists had not received any recent training in dementia (95.6%), suggesting a potential lack of awareness in how to best help people with dementia. Respondents also showed a lack of knowledge surrounding the struggles that people with dementia may have with their medication, for example swallowing oral dosage forms [29].

One study found that one healthcare professional felt it was important to support the informal carer directly, *“So that's where the help needs to be improved, empowering carers…You can't empower the patients because they're already losing them (to the symptoms of dementia)”* [28]. Additionally, one healthcare professional commented on the importance of clarity, stating that *“Written instructions, pictures and making sure that their…family understand what to do”* [28]. Further to this, one healthcare professional listed the support required for a person with dementia and their informal caregiver: *“Well, if they’re having problems taking it at the right time then I would say social services because they’d need prompting to take it. If it was because they couldn’t open bottles or they were getting pills mixed up, because you, like, you might get several tablets that look the same. You know, so that would be the pharmacist because you need identification. If it was because, like with PRN medication, you would maybe need a nurse to help them identify when they needed certain drugs.”* [28]. Similarly to this, one healthcare professional commented that care should be taken in the development of a medication regimen for people with dementia and their informal caregivers: *“If you’ve got a choice of inhaler but have these twice a day...or there’s one that’s once a day you’d, hopefully, go for the one that’s once a day one if it carries the appropriate medication. So, it’s just simplifying everything…get them the best medication possible, make it simple and then they are going to use it”* [28], thus demonstrating an application in practice to understanding and optimising medication in people with dementia.

Informal carers valued the partnership of healthcare professionals and themselves: *“If I didn’t have the relationship with the GP that I do, mum wouldn’t be at home; she’d be in a nursing home because I wouldn’t cope”* [12]. As well as this, values were placed on the collaborative partnership between healthcare professionals working together: *“The ideal was when the GP and pharmacist would work collaboratively”* [12]. However, there may be structural barriers to such collaborative working: “*I have no good contact with my mother’s GP. I cannot reach her normally, only by receptionist or by post”* [23].

## Discussion

As far as we are aware, this is the first literature review that has focussed on medicines management in people living in the community with dementia. Other reviews have included people living in care homes [14] and covered older people in general [8, 14]. Furthermore neither of these studies included the views of healthcare professionals or used an appraisal tool to assess the quality of the papers [8, 14]. Compared with these earlier reviews, there is limited data specifically focussed on medicines management in people living in the community with dementia. This current review also included the experiences of this population with healthcare professionals. People with dementia may lack the ability to understand and manage their medication. This increases the risk of non-adherence and results in informal carers taking on responsibility for medicines management as dementia progresses. This change in responsibility may affect the quality of life of the informal carer.

This review has identified the impact that medicines management responsibilities have on informal carers of people with dementia. The burden grew with increased complexity of the treatment regimen. This is reflected in other research that focused on general caregiving, not specifically for people with dementia [30] and not within the context of medicines management [31] and found informal carers often work out how to carry out their responsibilities through “trial and error”, due to the lack of prior knowledge or experience in the role [30, 31]. Similar to theme 2 looking at carer burden, research conducted on older adults has suggested carer burden may increase if informal caregivers have to balance administration of medication with other responsibilities [32]. Maintaining continuous supplies of medication, especially when informal carers have work commitments, and deciding if the care recipient was showing side effects from the medication have also been expressed as challenges [33]. This challenge was linked to informal carers struggling with interpreting information about the medication on the package inserts [33], which in the above review was shown to affect medication adherence (theme 2).

Overall medication adherence appears to be a challenge in people with dementia and so this group warrants further attention to develop ways of helping them continue to take their medication in a shared decision making model. Although the progressive nature of the disease creates difficulty for people with dementia to adhere correctly to their medication, it is still important to find ways for them to feel a part of the decision making process. This may involve adopting or adapting a shared decision making approach in the context of the cognitive abilities of the person with dementia.

Supporting medication adherence, especially with the use of adherence aids was an important concern found in this synthesis, especially in Themes 1 and 3. Adherence aids were one strategy used with varying degrees of effectiveness; these tended to only work in the early stages of the disease. Interventions targeted at providing education to caregivers on medicines management appear to be a sound basis for increased adherence. Teaching the care-recipient about medication could help with adherence [34]. However, educational interventions should consider the practical issues associated with administration faced by informal carers and support medicines management rather than solely focusing on adherence [35]. The training required for any educational intervention must be widely accessible for informal carers. Workshops where informal carers could meet and learn from each other may be helpful; however, some informal carers may not be able to leave their care-recipient unattended [30]. Not all informal carers may have the technological skills to use the internet [32]. Additionally, informal carers valued the chance to ask questions, which may be easier face-to-face.

This synthesis found that collaborative partnerships between informal carers and healthcare professionals were valued by informal caregivers and that healthcare professionals need to understand the varied role of informal carers (theme 4). Theme 4 also found that interventions to support medicines management for people with dementia should increase healthcare professionals’ awareness of the difficulties that informal carers face and the support that is available for them. Appropriately trained healthcare professionals should conduct regular medication reviews and ensure that their patients who have dementia are on the most straightforward routine available for them [36, 37]. A dementia training intervention, which included a physician-training program and support from local dementia service providers, assisted in improving dementia care in the primary care environment [38].

### Implications for healthcare professionals and policymakers

Healthcare professionals should be aware of the challenges faced by people with dementia and their informal carers and recognise the need for regular, on-going support [36]. However, as we found in theme 4, healthcare professionals may need specialist training in dementia care, especially in the responsibilities that informal carers face in looking after someone with dementia [13, 31, 39]. The role played by informal carers in the care of older adults has been formally recognised with the publication of new guidance for patient- and family-centred care [40]. Policy in this area should apply the principles of a patient-centred approach [40, 41]. Individualised care should be extended to support informal carers of people with dementia to enable sound medicines management without creating an excessive burden [41] a concern, which was highlighted in Theme 2.

### Limitations of the study

This review identified a relatively small number of studies, which demonstrates a lack of research conducted in the field both nationally and internationally. The results of this mixed studies review should be interpreted with caution due to the limitations of the included studies. Transparency of reporting is key to establishing the trustworthiness of findings and in turn improve the quality of the evidence base for future research and healthcare interventions [42]. Some studies in this review were appraised as poorer quality due to a lack of transparency in methods used [24, 27, 29]. Some of the included studies that adopted a qualitative methodology failed to adequately recognise and prevent researcher bias [12, 23, 24, 27]. The desire of carers to give the “correct response” to the interviewer may have affected the results [24]. There is also the potential of recall bias from the interviews conducted [26]. Some studies failed to adequately consider the impact of the context in which the data were collected on the findings [22], or had a low response rate affecting the generalisability of the results [29]. Triangulation with an independent data collection method, such as a daily diary entry of informal carers’ experiences of medicines management, may be a more appropriate way of understanding the challenges and may in turn help to reduce any researcher influences. In spite of these limitations, it should be noted that this review has brought together the current evidence and highlighted the areas that require additional focus through higher quality research.

### Future research

Further qualitative work should aim to understand more about how different groups of people with dementia and their carers manage their medication and the level of support they require. It would be beneficial to hear from a wide range of health and social care professionals, and understand their role in helping people with dementia manage their medication and the role of shared decision making in medication optimisation. Informed by the current evidence base and the future qualitative work an intervention to aid medicines management should be developed in line with the Medical Research Council framework for developing complex interventions [43].

## Conclusion

This mixed studies review identified that managing medication for people with dementia dwelling in the community is a complex task with a frequently associated burden on their informal caregivers. The risk of non-adherence exists even when the person with dementia is supported by an informal carer and even when adherence aids are used. Healthcare professionals may be unaware of this burden.

The need for informal carers to understand the prescribed medication and the benefits of taking it, as well as the need for support from healthcare professionals, was shown to be important to both the person with dementia and their informal carers. Healthcare professionals may need to undergo further training in supporting medicines management for people with dementia in their own homes. Overall, informal carers have a critical role in supporting medicines management in people with dementia and a patient-centred approach, to individual care, should be fully extended to enable informal carers to support people with dementia with their medication.
